# Chronic Pelvic Pain Syndrome in a Rural Area: Are We Paying Attention?

**DOI:** 10.7759/cureus.13525

**Published:** 2021-02-23

**Authors:** Omar S Akhtar

**Affiliations:** 1 Urology, Hakim Sanaullah Specialist Hospital, Sopore, IND; 2 Urology, Government Medical College, Srinagar, IND

**Keywords:** chronic prostatitis, chronic pelvic pain syndrome, lower urinary tract synptoms, prostatitis

## Abstract

Introduction

Chronic prostatitis/chronic pelvic pain syndrome (CP/CPPS) is a chronic, debilitating illness that mostly affects males under the age of 50 years. It is associated with myriad presentations. The National Institutes of Health-Chronic Prostatitis Symptom Index (NIH-CPSI) score was developed to measure the impact of the disease and assess the treatment outcomes. Additionally, the UPOINT [urinary (U), psychosocial (P), organ-specific (O), infection (I), neurologic/systemic (N), and tenderness of pelvic floor skeletal muscles (T)] classification system has been developed to enable a ‘phenotypic approach’ to the treatment.

Objective

The objective of this study was to determine the NIH-CPSI scores as well as the positive subdomain numbers and distribution of the UPOINT classification in patients with CP/CPPS.

Materials and methods

A total of 100 consecutive male patients presenting to a single centre with symptomatic CP/CPPS were included in this study.

Results

The mean age of the patients was 34.4 years. The average total NIH-CPSI score was 24.9. The average number of positive UPOINT domains was 2.28. The positive domains were urinary (90), psychosocial (60), organ-specific (43), infection (15), neurological (12), and tenderness (8).

Discussion

The NIH-CPSI scores and UPOINT subdomain scores compared favourably with other studies conducted in the region. The lower infection subdomain score as compared to other studies may be due to the widespread use of antibiotics among patients in the region prior to presenting to a urologist.

Conclusion

The use of the UPOINT classification to guide treatment is feasible even in a rural setting, such as in Kashmir.

## Introduction

Prostatitis-like symptoms are a common reason for outpatient visits to urologists among male patients under the age of 50 years, with a prevalence of 2-9% [1]. The National Institutes of Health (NIH) has classified prostatitis into four categories - type I: acute bacterial prostatitis; type II: chronic bacterial prostatitis; type III: chronic nonbacterial prostatitis or chronic pelvic pain syndrome (CNP/CPPS); and type IV: asymptomatic inflammatory prostatitis [2]. Type III is subclassified into IIIA (inflammatory) and IIIB (non-inflammatory), based on the presence of leucocytes in expressed prostatic secretions, urine specimen after the prostatic massage, or semen [2]. Chronic prostatitis type III or chronic pelvic pain syndrome (CP/CPPS) has a varied presentation but is most often characterised by genital pain, urinary problems, erectile and ejaculatory dysfunction, and psychological problems [3]. Erectile dysfunction (ED), which is the inability to develop and/or maintain an erection, is the most common sexual dysfunction reported along with CP/CPPS and has been reported in 46-92% of patients with CP/CPPS [4]. CP/CPPS is associated with myriad presentations, and different treatment methods, which necessitated a ‘phenotypic approach’ first advocated by Shoskes et al. in 2009 for the diagnosis and treatment of CP/CPPS. They proposed a ‘UPOINT’ classification or ‘Snowflake’ model, consisting of six subdomains of urinary, psychosocial, organ-specific, infection, neurological or systemic abnormalities, and muscle or skeletal tenderness (UPOINT) [5]. The UPOINT-directed treatment has been shown to significantly improve patient outcomes [6].

The study presented here is a prospective study of the use of the NIH-Chronic Prostatitis Symptom Index (NIH-CPSI) score and the UPOINT classification in a predominantly rural population of patients presenting to a single centre with features suggestive of CP/CPPS.

## Materials and methods

A total of 100 consecutive patients who met the criteria of CP/CPPS were examined by a single urologist in the urology outpatient department at the Hakim Sanaullah Specialist Hospital and Cancer Centre in Sopore, Kashmir, India, from April 2018 to December 2020. After a thorough history and physical examination, a two-glass test for urinary tract infection was performed to localise the infection to the prostate. This has been shown to be equivalent to the four-glass test [7]. All patients were evaluated according to the NIH-CPSI scores, with subscores determined based on pain (0-21 points), quality of life (QoL, 0-12 points), and urinary (0-10 points) scores (total score range: 0-43 points). Patients were classified into three domains: severe dysfunction (>29 points), moderate dysfunction (16-29 points), and mild dysfunction (0-15 points) according to their degree of symptoms as per the NIH-CPSI score. This method has been used previously in a study conducted in Turkey by Arda et al. [8]. The UPOINT framework was used to evaluate patients according to the relevant criteria. In all patients, at least one UPOINT subdomain positivity was present. Patients were specifically asked about their sexual history and the findings were recorded. All patients underwent a urinalysis, routine blood tests, and an ultrasound of the kidney, ureter, bladder, and prostate with the measurement of post-void urine volume and a pre- and post-prostatic massage urine culture.

The urinary subdomain was considered positive if there was high post-void residual volume (>100 ml, as measured by ultrasound), bothersome nocturia, urgency, and frequency of urination, or a CPSI urine subdomain score of ≥4. The psychosocial subdomain was determined to be positive if the patients stated that they were depressed and felt hopeless due to the disease. Organ-specific subdomain was considered to be positive if there was prostate tenderness upon rectal palpation, haematospermia, or sign of intense prostatic calcifications on ultrasound. The infection subdomain was deemed positive in patients other than in category I and II CP if growth was seen on urine culture on the second sample of the two-glass test. Neurological or systemic subdomain positivity was associated with pain outside of the pelvis or newly diagnosed irritable bowel syndrome, or chronic fatigue syndrome. Finally, the tenderness subdomain was considered positive if there was palpable muscle spasm or abdominopelvic trigger points. The phenotypic approach to diagnose patients was followed. Patients were treated using the multimodal treatment pathway as advocated by Shoskes et al. [6], as summarised in Table 1.

**Table 1:** Subdomain positivity based on Shoskes et al.* *[6] CPSI: Chronic Prostatitis Symptom Index; DRE: digital rectal examination

Domain	Clinical description
Urinary	CPSI score of >4
	Bothersome urgency, frequency, nocturia
	A flow rate of <15 ml/second, or obstructed pattern
	Post-void residue of >100 ml
Psychosocial	Depression
	Poor coping or maladaptive behavior, catastrophizing
Organ-specific	Prostate tenderness
	Haematospermia
	Leucocytosis in the prostatic secretion
	Extensive prostatic calcification
Infection	Excluded patients with evidence of acute or chronic bacterial prostatitis (recurrent - located to the prostate in repeated specimens)
	Gram-negative bacilli
	Documented response to antibacterial therapy
Neurologic/systemic conditions	Pain beyond pelvis
	Irritable bowel syndrome
	Chronic fatigue syndrome
Tenderness	Palpable tenderness and/or painful muscle spasm or trigger points in the perineum or pelvic floor or pelvic sidewalls during DRE

The exclusion criteria were as follows: patients with acute bacterial prostatitis, chronic bacterial prostatitis, history of genitourinary cancer or genitourinary tuberculosis, urethral stricture, history of recent endourological procedure, or a diagnosis of neurogenic bladder.

Statistical analysis

Data were analysed statistically using the available software in Microsoft Excel (Office 365, Microsoft Corporation, Redmond, WA), and Google Drive (Google, Alphabet Inc., Mountain View, CA). The UPOINT and NIH-CPSI scores of patients were analysed. Data were presented as means and standard deviations. A p-value of <0.05 was considered statistically significant.

## Results

 A total of 100 consecutive patients were enrolled in the study. Demographic data of the subjects are presented in Table 2.

**Table 2:** Demographic profile of patients SD: standard deviation; NIH-CPSI: National Institutes of Health-Chronic Prostatitis Symptom Index; QoL: quality of life; IPSS: International Prostate Symptom Score

Variables	Values
Mean age, years (range, SD)	34.37 (21-60, 8.55)
Residence type, n (%)	Rural: 98/100 (98%); urban 2/100 (2%)
Profession, n (%)	Salaried: 11/100 (11%); daily-wage earner: 34/100 (34%); self-employed: 10/100 (10%); farmer/orchardist: 45/100 (45%)
Average duration of symptoms, months (range, SD)	8 (3-24, 5.01)
NIH-CPSI scores	
Total, average (range, SD)	24.9 (14-34, 5.06)
Pain, average (range, SD)	11.4 (4-18, 3.49)
Voiding, average (range, SD)	6.3 (2-8, 1.85)
QoL, average (range, SD)	7.2 (4-11, 2.14)
IPSS, average (range, SD)	9.4 (4-26, 4.74)
Pain severity, n (%)	
Mild	10 (10%)
Moderate	66 (66%)
Severe	24 (24%)

**Figure 1:**
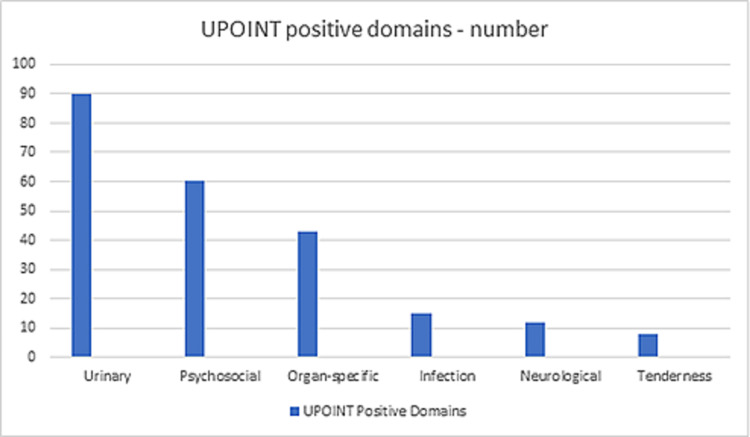
Positive domains among patients UPOINT: urinary (U), psychosocial (P), organ-specific (O), infection (I), neurologic/systemic (N), and tenderness of pelvic floor skeletal muscles (T)

**Figure 2:**
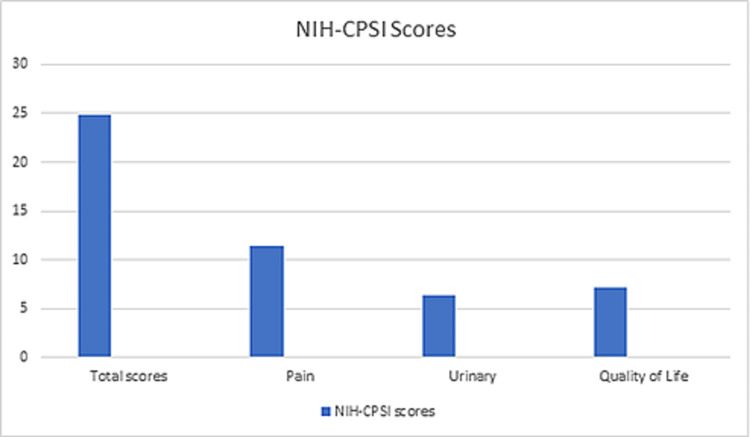
NIH-CPSI scores NIH-CPSI: National Institutes of Health-Chronic Prostatitis Symptom Index

The NIH-CPSI and UPOINT subdomain data of these patients were analysed. The average total NIH-CPSI score was 24.9 (range: 14-34, SD: 5.06) and the average positive UPOINT subdomain number was 2.28 (Table 3, Figure 1). Positive subdomains were determined as follows - urinary: 90 (90%); psychosocial: 60 (60%); organ-specific: 43 (43%); infection: 15 (15%); neurological or systemic: 12 (12%); and tenderness: 8 (8%). For validating the UPOINT and NIH-CPSI scores, we calculated the correlation between positive UPOINT subdomain number and NIH-CPSI total score and subdomain pain (0-21 points), QoL (0-12 points), and urinary (0-10 points) scores in the patient population (Figure 2). A correlation was seen between UPOINT subdomain positivity and NIH-CSPI total score. When the subdomains urinary, pain, and QoL were considered separately, a correlation with UPOINT subdomain positivity was observed. It was also observed that as the UPOINT subdomain number positivity increased, there was a gradual increase in the NIH-CPSI score.

**Table 3:** UPOINT positive domains UPOINT: urinary (U), psychosocial (P), organ-specific (O), infection (I), neurologic/systemic (N), and tenderness of pelvic floor skeletal muscles (T)

Domain (percentage positive)	Number (%) of positive scores
Urinary	90 (90%)
Psychosocial	60 (60%)
Organ-specific	43 (43%)
Infection	15 (15%)
Neurological/systemic	12 (12%)
Skeletal muscle tenderness	8 (8%)

The total number of patients with CP/CPPS constituted 7% of the total urology outpatient visits during the study period (Table 4).

**Table 4:** CP/CPPS patients out of total urology outpatient visits CP/CPPS: chronic prostatitis/chronic pelvic pain syndrome

Urology outpatient department visits	
Total	1,418
CP/CPPS diagnosis	100 (7%)

## Discussion

CP/CPPS remains a common condition that impacts many males under the age of 50 years [9]. Wagenlehner et al. have shown that a classification-directed approach could improve the treatment outcomes of this disease since it has a varied presentation [10]. Shoskes et al. came up with the UPOINT classification, which is a phenotypic approach to CP/CPPS, in the same year [5]. However, an effective treatment method for CP/CPPS remains elusive, and some studies have yielded negative or conflicting results [11-13]. Many patients have reported spending a considerable amount of time for clinic visits and money on medications, without achieving any resolution of their symptoms, which is an important consideration in our study population, a significant proportion of whom are rural and daily-wage earners [14]. Multimodal therapy based on the patient’s UPOINT phenotype has been reported to achieve impressive results [8].

Our study showed a correlation between NIH-CPSI scores and the number of positive domains in the UPOINT score, which was in line with the findings of Shoskes et al. [5]. The number of positive UPOINT domains correlated with the symptom severity as measured by NIH‑CPSI scores. There was an increase in the NIH‑CPSI total score, pain subscore, urinary subscore, and Qol subscore as the number of positive UPOINT domains increased.

In this study, the average duration of symptoms among patients before presentation to the urologist was eight months. This observation validates the hypothesis that ongoing local inflammation and coincident tissue injury can cause local muscle spasms, with the central nervous system and peripheral nervous system alterations, and can cause psychosocial changes that can maintain the symptoms for many months after the initial insult has passed [13].

The mean age of the patients in our study was 34.4 years. The prevalence and the distribution of positive UPOINT domains and the NIH-CPSI scores in our patients were similar to those described in the Chinese [14,15] and Turkish [8] studies on CP/CPPS. The similarity of results between different patient populations in different countries and health systems underlines the usefulness of the UPOINT phenotype system. Although the study sample was small, the results of our study among Kashmiri patients confirm the utility of the UPOINT algorithm for classifying CP/CPPS.

The number of patients who presented with positive Infection subdomain scores (15, 15%) was less compared to other studies [8,14,15]. This is probably a result of the widespread use of antibiotics in the region. It is plausible that many patients who had mild urinary tract or prostatic infections had already been treated for their infections, and had become culture-negative before presenting to the study centre.

Our study was not designed to evaluate the results of multimodal therapy, as has been done in other studies [15]. However, we were able to use the UPOINT phenotype to direct multimodal treatment successfully, as opposed to monotherapy previously.

The limitations of this study include the smaller sample size compared to other studies and the fact that this was a single-centre study. This study was conducted in a rural centre. Many patients had been seen and cared for by other physicians prior to their presentation to our hospital. Hence, many patients with less severe symptoms may have been left out. The study was not designed to include treatment outcomes, and we did not include a sexual-dysfunction domain in our analysis.

## Conclusions

Based on our findings, the UPOINT classification system and the NIH-CPSI score can be effectively used in a predominantly rural population to evaluate and direct multimodal treatment for CP/CPPS. Multi-centric studies are required to further analyse the UPOINT system and NIH-CPSI scores, and to assess multimodal treatment outcomes and the relationship of CP/CPPS with sexual dysfunction within this population.
